# Supplementary material for: Genotypic Effect on Olive (Olea europaea) Fruit Phenolic Profile
Source: Plants (Basel). 2025 Jun 28;14(13):1981. doi: 10.3390/plants14131981 (PMC12251559; doi:10.3390/plants14131981)
Supplement: Supplementary file 1 [file plants-14-01981-s001.zip › plants-3699670-supplementary.pdf]

| Genotype | TOTAL | DERHT | HT1G | HT4G | DMOLEU |
|---|---|---|---|---|---|
| Abbadi Abou Gabra842 | 8116,52 | 7508,78 | 1128,12 | 147,66 | 14,63 |
| Abou Kanani | 13950,05 | 13272,55 | 1661,40 | 71,82 | 22,56 |
| Abou Salt Mohazam | 7533,96 | 6638,52 | 477,02 | 45,71 | 18,43 |
| AcebR1048 | 40217,46 | 30936,39 | 853,30 | 1784,36 | 4263,42 |
| AcebR121 | 17702,99 | 14583,17 | 473,09 | 770,41 | 1554,98 |
| AcebR183 | 16859,12 | 15706,43 | 982,32 | 407,11 | 12171,21 |
| AcebR224 | 38215,95 | 32624,58 | 1795,40 | 1971,42 | 20066,62 |
| AcebR225 | 28021,45 | 24718,22 | 1682,97 | 530,28 | 8818,83 |
| AcebR302 | 64924,12 | 55068,43 | 1128,05 | 629,64 | 21445,21 |
| AcebR304 | 74999,68 | 68289,81 | 479,33 | 647,22 | 17094,07 |
| AcebR37 | 42240,83 | 36158,08 | 332,57 | 965,86 | 11876,38 |
| AcebR74 | 33672,06 | 27384,19 | 459,95 | 972,54 | 9247,12 |
| AcebR78 | 25121,18 | 22686,83 | 689,90 | 2308,31 | 11116,88 |
| Adramitini/Ayvalik | 11634,74 | 10471,22 | 2218,31 | 112,07 | 56,98 |
| Aggizi Shame | 6035,30 | 5575,63 | 536,32 | 151,01 | 11,77 |
| Aloreña | 5693,80 | 5081,22 | 1352,49 | 63,99 | 5,54 |
| Arbequina | 10888,37 | 10097,23 | 718,14 | 78,51 | 6399,61 |
| Arbosana | 11192,85 | 10274,22 | 1037,54 | 57,40 | 4967,73 |
| Ascolana Tenera | 10283,81 | 9437,03 | 2965,58 | 901,92 | 13,55 |
| Azapa | 15411,00 | 13447,64 | 792,63 | 366,54 | 1981,25 |
| Baladi | 10551,50 | 9073,83 | 219,89 | 5,96 | 16,66 |
| Barnea | 9648,13 | 8622,43 | 260,90 | 9,18 | 18,71 |
| Barri | 18894,09 | 16730,15 | 353,52 | 209,53 | 41,53 |
| Calatina | 14398,27 | 13600,60 | 2109,41 | 188,45 | 9,07 |
| Changlot Real | 14044,78 | 12521,67 | 1150,92 | 249,72 | 17,05 |
| Chemlal de Kabilye | 13335,97 | 11665,49 | 1104,82 | 218,84 | 8,06 |
| Chemlali Sfax | 14566,85 | 13322,15 | 833,12 | 92,50 | 231,82 |
| Coratina | 26231,38 | 23657,51 | 128,50 | 39,92 | 2109,63 |
| Dahgan | 10234,48 | 9232,23 | 1066,35 | 64,17 | 5,62 |
| Dokkar | 26464,03 | 24773,98 | 971,94 | 963,03 | 16620,60 |
| Empeltre | 10672,56 | 9963,52 | 833,46 | 52,53 | 7222,22 |
| Farga | 7840,42 | 7165,15 | 2495,31 | 982,39 | 57,65 |
| Fishomi | 7580,64 | 6604,27 | 811,64 | 118,77 | 15,95 |
| Forastera de Tortosa | 8021,64 | 7127,22 | 1820,78 | 137,22 | 4013,82 |
| Frantoio | 10476,36 | 9431,74 | 489,45 | 89,53 | 4129,87 |
| Galega Vulgar | 14364,50 | 13843,96 | 654,43 | 333,71 | 10770,46 |
| Gaydorelia/Esek zeytini (Odemis) | 9834,18 | 9438,74 | 690,06 | 111,85 | 15,32 |
| Gemlik | 8233,37 | 7486,28 | 513,24 | 14,03 | 19,64 |
| Gordal Sevillana/Tavsan yuregi | 4390,84 | 4047,71 | 1306,55 | 58,31 | 13,26 |
| Grappolo | 11501,44 | 10498,89 | 132,22 | 6,67 | 3585,10 |
| Habichuelero de Grazalema | 18221,59 | 15496,33 | 1091,32 | 270,76 | 37,37 |
| Hojiblanca | 10135,99 | 9267,23 | 3185,64 | 83,68 | 15,00 |
| Jabali | 6444,58 | 5774,60 | 917,82 | 270,50 | 21,54 |
| Kalamon | 13017,43 | 10904,04 | 2506,11 | 329,89 | 28,36 |
| Klon-14-1812 | 14427,66 | 12722,54 | 210,91 | 16,00 | 9,28 |
| Konservolia/Cizmelik (Tekirdag) | 6678,16 | 5899,37 | 501,72 | 40,26 | 10,82 |
| Koroneiki | 13809,84 | 12442,05 | 378,51 | 159,32 | 13,75 |
| Koutsourelia | 18387,59 | 16182,11 | 803,28 | 305,18 | 10,63 |
| Leccino | 13464,36 | 12121,28 | 434,79 | 319,19 | 8427,48 |

| | | | | | |
|---|---|---|---|---|---|
| Lechín de Granada/Dafnelia | 20930,46 | 18916,00 | 1140,94 | 65,65 | 7,94 |
| Lechín de Sevilla | 11055,05 | 10225,90 | 2809,08 | 669,80 | 26,07 |
| Lianolia Kerkyras | 23475,59 | 21520,45 | 823,53 | 173,95 | 1581,91 |
| Llumeta | 9066,97 | 7719,99 | 1044,98 | 586,25 | 34,10 |
| Maarri | 7921,51 | 6700,79 | 351,92 | 29,28 | 10,89 |
| Majhol-152 | 21527,58 | 19985,01 | 2189,65 | 59,31 | 13,72 |
| Manzanilla Aceitera | 20454,68 | 19210,50 | 823,51 | 711,71 | 5410,28 |
| Manzanilla Cacereña | 6167,31 | 5406,49 | 1587,55 | 203,18 | 12,87 |
| Manzanilla de Sevilla | 14792,30 | 13244,89 | 746,93 | 125,33 | 8,35 |
| Manzanillera de Huércal Overa | 8398,71 | 7106,24 | 447,78 | 180,68 | 16,66 |
| Mari | 13161,98 | 10712,36 | 711,68 | 363,36 | 12,01 |
| Mastoidis | 17303,13 | 15236,80 | 532,68 | 27,10 | 22,11 |
| Mavrelia | 10048,34 | 9086,26 | 498,05 | 39,25 | 17,38 |
| Mawi | 15054,52 | 14081,19 | 210,80 | 62,98 | 10,24 |
| Menya | 18271,26 | 16571,19 | 590,32 | 183,59 | 7836,28 |
| Moraiolo | 11501,38 | 10741,00 | 2273,95 | 343,16 | 9,31 |
| Morrut | 17293,72 | 16346,62 | 473,50 | 96,78 | 16,26 |
| Nabali/ Gordal de Granada | 9570,47 | 8761,78 | 582,40 | 131,06 | 19,56 |
| Ocal/Bouchouk laghid | 9345,05 | 8481,17 | 1659,41 | 516,80 | 33,00 |
| Pajarero | 8904,39 | 7579,55 | 657,50 | 22,60 | 16,70 |
| Picholine Marocaine | 16703,08 | 15477,86 | 4457,61 | 200,91 | 30,81 |
| Picholine du Languedoc/Abou Chawkeh | 23676,73 | 21424,88 | 1240,44 | 282,88 | 24,06 |
| Picual | 13750,40 | 12617,46 | 472,06 | 65,38 | 10,57 |
| Picudo | 14950,86 | 12976,10 | 1071,19 | 555,95 | 30,31 |
| Piñonera | 15370,43 | 13814,26 | 789,74 | 142,92 | 7716,89 |
| Royal de Cazorla | 7701,93 | 7379,10 | 2655,51 | 66,81 | 33,43 |
| Royeta de Asque | 7941,56 | 7033,03 | 2482,51 | 209,29 | 7,10 |
| Safrawi | 17259,41 | 15161,52 | 1410,53 | 520,31 | 16,52 |
| Saifi/Baladi (Fekhe) | 5315,58 | 4732,99 | 1657,52 | 621,45 | 14,65 |
| Sikitita | 9928,76 | 8959,09 | 1208,49 | 106,28 | 23,41 |
| Temprano | 8343,21 | 7848,58 | 720,53 | 72,40 | 1740,45 |
| Ulliri i Bardhe i Tiranes | 9815,00 | 8980,58 | 4425,14 | 263,09 | 20,59 |
| Uovo di Piccione | 14043,94 | 11834,02 | 900,85 | 632,24 | 27,80 |
| Uslu | 7732,33 | 7033,95 | 1226,31 | 27,49 | 15,89 |
| Verdial Velez-Málaga-51 | 5917,30 | 5145,09 | 1433,29 | 51,07 | 12,16 |
| Verdial de Badajoz | 8749,91 | 7649,27 | 522,77 | 56,57 | 24,03 |

| OLEU | AGOLEU | VERBAS | DERTY | TY1G | DMLIGS | LIGS | AGLIGS |
|---|---|---|---|---|---|---|---|
| 5352,89 | 4,27 | 861,22 | 346,17 | 142,15 | 12,34 | 184,68 | 6,99 |
| 11350,30 | 4,01 | 162,46 | 237,08 | 68,66 | 11,56 | 147,23 | 9,62 |
| 5593,08 | 368,40 | 135,89 | 402,57 | 74,66 | 40,06 | 254,89 | 32,96 |
| 21335,04 | 1327,82 | 1372,45 | 6008,12 | 2141,49 | 701,64 | 2966,53 | 198,46 |
| 9724,06 | 526,26 | 1534,38 | 2510,83 | 965,03 | 384,26 | 1129,73 | 31,80 |
| 96,06 | 4,66 | 2045,07 | 487,89 | 229,73 | 226,26 | 28,65 | 3,24 |
| 6669,03 | 740,63 | 1381,48 | 3212,49 | 1202,38 | 1649,58 | 332,43 | 28,10 |
| 11598,93 | 978,66 | 1108,55 | 2284,24 | 612,56 | 499,75 | 1071,77 | 100,17 |
| 29337,83 | 2124,25 | 403,46 | 7793,59 | 863,88 | 2894,47 | 3678,65 | 356,59 |
| 43086,10 | 4851,21 | 2131,88 | 6089,58 | 332,00 | 1730,29 | 3610,09 | 417,19 |
| 16918,34 | 2977,05 | 3087,88 | 4667,85 | 755,49 | 1486,37 | 2049,36 | 376,63 |
| 13998,80 | 779,62 | 1926,17 | 4677,97 | 1018,88 | 1419,85 | 2049,03 | 190,21 |
| 5235,03 | 846,31 | 2490,39 | 2186,20 | 1314,78 | 594,46 | 226,39 | 50,57 |
| 7192,42 | 9,66 | 881,78 | 180,81 | 38,32 | 20,67 | 113,83 | 7,99 |
| 4839,17 | 6,21 | 31,15 | 149,07 | 35,89 | 19,77 | 86,45 | 6,96 |
| 2772,20 | 5,13 | 881,87 | 313,95 | 129,48 | 13,92 | 164,14 | 6,41 |
| 2394,56 | 5,40 | 501,02 | 345,18 | 60,94 | 193,56 | 82,51 | 8,16 |
| 3397,45 | 10,69 | 803,41 | 383,71 | 41,16 | 209,35 | 124,56 | 8,64 |
| 3030,53 | 7,61 | 2517,86 | 680,52 | 587,36 | 24,45 | 60,03 | 8,68 |
| 10007,35 | 45,74 | 254,13 | 1436,31 | 180,96 | 88,88 | 1147,48 | 18,98 |
| 8786,46 | 7,88 | 36,99 | 1034,07 | 38,04 | 14,83 | 973,51 | 7,69 |
| 8142,27 | 56,53 | 134,85 | 682,11 | 25,97 | 23,32 | 621,41 | 11,39 |
| 13745,47 | 36,60 | 2343,51 | 1220,40 | 86,70 | 64,06 | 1058,14 | 11,52 |
| 10612,52 | 329,85 | 351,31 | 420,22 | 112,24 | 48,34 | 250,06 | 9,59 |
| 9024,16 | 10,01 | 2069,83 | 1340,45 | 389,87 | 29,35 | 905,56 | 15,67 |
| 8062,10 | 460,26 | 1811,40 | 1310,01 | 140,40 | 24,70 | 1077,96 | 66,94 |
| 11713,53 | 51,06 | 400,12 | 746,86 | 25,85 | 39,93 | 674,10 | 6,99 |
| 17972,67 | 238,39 | 3168,39 | 2124,47 | 39,27 | 218,56 | 1843,53 | 23,11 |
| 7957,02 | 86,02 | 53,06 | 758,78 | 114,18 | 24,51 | 612,25 | 7,84 |
| 4336,17 | 260,28 | 1621,95 | 1404,22 | 341,04 | 899,98 | 130,38 | 32,81 |
| 1407,23 | 38,49 | 409,61 | 349,17 | 61,18 | 234,22 | 44,22 | 9,54 |
| 3499,71 | 6,92 | 123,17 | 386,12 | 342,23 | 10,70 | 25,45 | 7,73 |
| 5556,69 | 9,40 | 91,82 | 541,34 | 107,89 | 19,80 | 398,59 | 15,06 |
| 124,51 | 8,39 | 1022,49 | 369,01 | 259,27 | 85,86 | 16,09 | 7,79 |
| 3347,63 | 4,62 | 1370,65 | 609,76 | 73,10 | 309,62 | 218,07 | 8,96 |
| 1750,91 | 7,03 | 327,41 | 372,12 | 88,14 | 241,87 | 35,56 | 6,54 |
| 7724,17 | 6,89 | 890,46 | 276,61 | 37,40 | 17,94 | 216,53 | 4,74 |
| 6691,29 | 8,19 | 239,89 | 442,01 | 16,35 | 52,24 | 365,12 | 8,30 |
| 2262,64 | 5,98 | 400,97 | 250,74 | 91,86 | 21,17 | 125,66 | 12,05 |
| 6540,53 | 2,78 | 231,58 | 570,21 | 71,28 | 150,78 | 339,22 | 8,92 |
| 12934,57 | 12,84 | 1149,48 | 1724,05 | 127,62 | 19,30 | 1558,84 | 18,29 |
| 4397,72 | 7,06 | 1578,13 | 306,33 | 58,88 | 49,40 | 190,30 | 7,74 |
| 4429,50 | 31,39 | 103,86 | 361,34 | 131,32 | 20,61 | 199,86 | 9,55 |
| 6323,55 | 11,32 | 1704,81 | 1468,26 | 668,68 | 42,28 | 746,90 | 10,40 |
| 12451,85 | 5,72 | 28,78 | 950,81 | 19,41 | 11,28 | 915,43 | 4,68 |
| 5282,06 | 4,07 | 60,44 | 553,38 | 119,69 | 25,09 | 396,95 | 11,65 |
| 11560,97 | 14,42 | 315,08 | 694,67 | 47,33 | 10,51 | 625,21 | 11,63 |
| 13944,11 | 4,58 | 1114,32 | 980,85 | 84,55 | 9,88 | 879,30 | 7,11 |
| 1825,56 | 5,94 | 1108,32 | 461,20 | 90,19 | 294,32 | 71,29 | 5,40 |

| | | | | | | | |
|---|---|---|---|---|---|---|---|
| 16674,73 | 49,36 | 977,39 | 1109,22 | 48,02 | 17,26 | 1031,14 | 12,80 |
| 4655,22 | 6,11 | 2059,62 | 737,42 | 466,56 | 30,17 | 231,81 | 8,88 |
| 15466,57 | 190,71 | 3283,79 | 1304,52 | 102,61 | 55,86 | 1125,60 | 20,45 |
| 5994,13 | 3,68 | 56,84 | 1147,03 | 603,39 | 39,19 | 495,66 | 8,79 |
| 6180,78 | 32,71 | 95,19 | 783,01 | 33,16 | 25,84 | 715,28 | 8,73 |
| 16594,23 | 940,94 | 187,16 | 721,69 | 91,15 | 89,04 | 514,89 | 26,62 |
| 8391,65 | 7,18 | 3866,18 | 916,94 | 76,04 | 337,54 | 496,50 | 6,86 |
| 3286,72 | 17,91 | 298,25 | 517,77 | 320,10 | 36,03 | 144,62 | 17,02 |
| 11920,00 | 6,61 | 437,67 | 1138,40 | 96,19 | 17,55 | 1017,54 | 7,12 |
| 5907,79 | 139,75 | 413,58 | 866,52 | 254,34 | 27,94 | 566,69 | 17,54 |
| 9448,38 | 69,38 | 107,54 | 567,33 | 225,50 | 36,61 | 297,02 | 8,21 |
| 12691,17 | 9,66 | 1954,07 | 1109,09 | 46,55 | 15,98 | 1040,47 | 6,10 |
| 7930,14 | 9,30 | 592,14 | 542,34 | 63,12 | 20,52 | 452,65 | 6,05 |
| 12565,40 | 806,73 | 425,04 | 620,69 | 52,42 | 41,22 | 501,75 | 25,30 |
| 7618,28 | 49,03 | 293,70 | 1308,89 | 99,40 | 498,60 | 696,80 | 14,09 |
| 5604,94 | 4,89 | 2504,75 | 203,49 | 59,99 | 13,20 | 121,17 | 9,14 |
| 13583,26 | 9,49 | 2167,33 | 628,85 | 68,02 | 26,23 | 520,65 | 13,95 |
| 7597,54 | 7,64 | 423,60 | 508,79 | 63,19 | 19,98 | 419,53 | 6,09 |
| 6228,95 | 5,81 | 37,20 | 541,58 | 227,22 | 54,74 | 250,46 | 9,16 |
| 6595,10 | 8,80 | 278,86 | 819,25 | 49,21 | 23,30 | 735,45 | 11,30 |
| 9120,39 | 216,51 | 1451,63 | 786,71 | 405,58 | 35,82 | 337,05 | 8,26 |
| 16898,45 | 119,81 | 2859,23 | 1807,78 | 256,32 | 42,90 | 1485,37 | 23,19 |
| 11584,46 | 5,77 | 479,22 | 612,82 | 34,12 | 16,73 | 551,80 | 10,17 |
| 11068,19 | 6,57 | 243,88 | 1038,26 | 372,58 | 20,65 | 632,88 | 12,15 |
| 4115,97 | 146,30 | 902,44 | 974,53 | 177,95 | 524,55 | 258,57 | 13,45 |
| 4442,37 | 4,66 | 176,33 | 148,90 | 56,92 | 26,66 | 58,70 | 6,62 |
| 3720,08 | 4,37 | 609,68 | 245,43 | 150,31 | 9,57 | 81,82 | 3,73 |
| 13007,69 | 8,68 | 197,79 | 1005,53 | 349,71 | 31,61 | 615,16 | 9,05 |
| 2342,15 | 8,64 | 88,57 | 347,90 | 233,04 | 27,05 | 82,81 | 5,00 |
| 6930,25 | 8,03 | 682,62 | 336,36 | 53,28 | 22,36 | 250,90 | 9,82 |
| 5065,17 | 5,76 | 244,27 | 191,41 | 44,41 | 34,54 | 107,81 | 4,65 |
| 3558,46 | 4,95 | 708,36 | 597,99 | 429,20 | 16,43 | 145,31 | 7,05 |
| 7884,77 | 11,39 | 2376,96 | 1687,77 | 307,41 | 19,95 | 1346,70 | 13,71 |
| 5197,16 | 70,54 | 496,56 | 159,64 | 35,44 | 19,01 | 98,36 | 6,83 |
| 3552,08 | 3,76 | 92,73 | 362,41 | 136,12 | 28,67 | 188,53 | 9,08 |
| 6533,13 | 4,60 | 508,17 | 846,03 | 50,92 | 28,45 | 759,70 | 6,96 |

| FLV | RUT | LUT7G | API7G |
|---|---|---|---|
| 261,57 | 126,31 | 129,14 | 6,13 |
| 440,43 | 231,87 | 202,44 | 6,11 |
| 492,87 | 297,86 | 180,74 | 14,27 |
| 3272,95 | 2147,07 | 598,97 | 526,91 |
| 608,99 | 397,41 | 158,38 | 53,20 |
| 664,80 | 496,50 | 143,84 | 24,45 |
| 2378,88 | 1593,95 | 574,29 | 210,64 |
| 1018,98 | 690,72 | 288,75 | 39,51 |
| 2062,10 | 1115,51 | 852,79 | 93,80 |
| 620,29 | 503,69 | 84,85 | 31,76 |
| 1414,90 | 1188,01 | 154,94 | 71,95 |
| 1609,90 | 1125,36 | 317,80 | 166,75 |
| 248,15 | 172,33 | 67,40 | 8,41 |
| 982,72 | 545,32 | 401,72 | 35,68 |
| 310,60 | 184,63 | 117,11 | 8,86 |
| 298,63 | 137,35 | 154,81 | 6,47 |
| 445,96 | 226,64 | 204,26 | 15,06 |
| 534,91 | 307,59 | 205,11 | 22,21 |
| 166,26 | 108,10 | 55,03 | 3,13 |
| 527,04 | 275,13 | 224,14 | 27,77 |
| 443,61 | 280,84 | 153,05 | 9,72 |
| 343,60 | 197,20 | 133,13 | 13,27 |
| 943,54 | 470,18 | 429,93 | 43,42 |
| 377,44 | 251,19 | 121,06 | 5,19 |
| 182,66 | 77,97 | 96,70 | 7,99 |
| 360,48 | 246,74 | 107,96 | 5,77 |
| 497,83 | 370,90 | 106,87 | 20,06 |
| 449,40 | 297,25 | 142,08 | 10,07 |
| 243,47 | 133,69 | 106,08 | 3,69 |
| 285,83 | 230,02 | 46,78 | 9,04 |
| 359,87 | 244,73 | 101,80 | 13,34 |
| 289,16 | 210,92 | 67,97 | 10,26 |
| 435,03 | 292,20 | 123,47 | 19,36 |
| 525,41 | 369,82 | 149,74 | 5,84 |
| 434,86 | 273,02 | 150,33 | 11,52 |
| 148,42 | 106,51 | 38,02 | 3,88 |
| 118,83 | 44,60 | 67,06 | 7,17 |
| 305,08 | 139,67 | 148,71 | 16,69 |
| 92,39 | 53,67 | 35,60 | 3,12 |
| 432,34 | 276,65 | 143,69 | 12,01 |
| 1001,21 | 686,28 | 302,88 | 12,05 |
| 562,44 | 233,12 | 295,45 | 33,87 |
| 308,63 | 186,66 | 114,28 | 7,69 |
| 645,14 | 394,24 | 239,34 | 11,55 |
| 754,31 | 467,59 | 268,73 | 17,99 |
| 225,40 | 128,15 | 93,06 | 4,20 |
| 673,12 | 506,46 | 153,36 | 13,30 |
| 1224,64 | 672,84 | 510,60 | 41,20 |
| 881,88 | 601,63 | 266,67 | 13,58 |

| | | | |
|---|---|---|---|
| 905,24 | 562,53 | 315,85 | 26,87 |
| 91,73 | 66,62 | 21,23 | 3,88 |
| 650,62 | 456,37 | 174,57 | 19,68 |
| 199,96 | 138,49 | 53,10 | 8,36 |
| 437,72 | 259,76 | 169,13 | 8,83 |
| 820,88 | 434,69 | 352,62 | 33,56 |
| 327,23 | 190,76 | 126,09 | 10,39 |
| 243,05 | 138,15 | 97,29 | 7,61 |
| 409,01 | 241,59 | 155,84 | 11,59 |
| 425,96 | 258,66 | 149,06 | 18,24 |
| 1882,28 | 1159,44 | 653,30 | 69,55 |
| 957,24 | 662,45 | 265,64 | 29,15 |
| 419,74 | 256,85 | 153,08 | 9,81 |
| 352,64 | 215,32 | 126,40 | 10,92 |
| 391,18 | 279,43 | 98,85 | 12,90 |
| 556,89 | 368,25 | 173,03 | 15,61 |
| 318,26 | 236,42 | 66,86 | 14,99 |
| 299,90 | 145,97 | 148,17 | 5,76 |
| 322,30 | 166,68 | 148,38 | 7,24 |
| 505,59 | 350,29 | 138,90 | 16,40 |
| 438,51 | 328,20 | 98,95 | 11,35 |
| 444,07 | 249,18 | 182,09 | 12,80 |
| 520,11 | 291,07 | 211,18 | 17,86 |
| 936,50 | 596,00 | 308,76 | 31,74 |
| 581,64 | 488,44 | 85,40 | 7,80 |
| 173,93 | 120,30 | 45,61 | 8,02 |
| 663,10 | 368,22 | 283,34 | 11,54 |
| 1092,36 | 630,98 | 410,08 | 51,30 |
| 234,69 | 108,37 | 118,14 | 8,18 |
| 633,32 | 350,84 | 257,12 | 25,35 |
| 303,22 | 165,67 | 122,52 | 15,03 |
| 236,42 | 130,02 | 95,52 | 10,89 |
| 522,15 | 358,75 | 152,26 | 11,13 |
| 538,73 | 240,44 | 255,27 | 43,02 |
| 409,80 | 243,11 | 142,26 | 24,42 |
| 254,61 | 165,78 | 84,24 | 4,58 |
